# Functional traits of broad-leaved monocot herbs in the understory and forest edges of a Costa Rican rainforest

**DOI:** 10.7717/peerj.9958

**Published:** 2020-10-27

**Authors:** Philip W. Rundel, Arielle M. Cooley, Katharine L. Gerst, Erin C. Riordan, M. Rasoul Sharifi, Jennifer W. Sun, J. Alexandra Tower

**Affiliations:** 1 Ecology and Evolutionary Biology, UCLA, Los Angeles, CA, United States of America; 2 Biology, Whitman College, Walla Walla, WA, United States of America; 3 USA National Phenlogical Network, University of Arizona, Tucson, AZ, United States of America; 4 Laboratory of Tree Ring Research, University of Arizona, Tucson, AZ, United States of America; 5 Biology, Santa Monica College, Santa Monica, CA, United States of America

**Keywords:** Understory, Forest edge, Tropical forest, Araceae, Costaceae, Marantaceae, Zingiberaceae, Cyclanthaceae

## Abstract

Broad-leaved monocot herbs are widespread and dominant components of the shaded understories of wet neotropical forests. These understory habitats are characterized by light limitation and a constant threat of falling branches. Many shaded understory herb species have close relatives that occupy forest edges and gaps, where light availability is higher and defoliation threat is lower, creating an opportunity for comparative analysis of functional traits in order to better understand the evolutionary adaptations associated with this habitat transition. We documented ecological, morphological and ecophysiological traits of multiple herb species in six monocot families from each of these two habitats in the wet tropical rainforest at the La Selva Biological Station, Costa Rica. We found that a mixture of phylogenetic canalization and ecological selection for specific habitats helped explain patterns of functional traits. Understory herbs were significantly shorter and had smaller leaves than forest edge species. Although the mean number of leaves per plant and specific leaf area did not differ between the two groups, the larger leaf size of forest edge species gave them more than three times the mean plant leaf area. Measures of leaf water content and nitrogen content varied within both groups and mean values were not significantly different. Despite the high leaf nitrogen contents, the maximum photosynthetic rates of understory herbs were quite low. Measures of *δ*^13^C as an analog of water use efficiency found significantly lower (more negative) values in understory herbs compared to forest edge species. Clonality was strongly developed in several species but did not show strong phylogenetic patterns. This study highlights many functional traits that differ between broad-leaved monocot species characteristic of understory and forest edge habitats, as well as traits that vary primarily by phylogenetic relatedness. Overall, plant functional traits do not provide a simple explanation for the relative differences in abundance for individual understory and forest edge species with some occurring in great abundance while others are relatively rare.

## Introduction

Although trees form the focus of much of the literature on tropical forests, there are many other significant growth forms that collectively often represent a greater species diversity (Gentry & Dodson, 1987; Poulsen & Balslev, 1991; Linares-Palomino et al., 2009). These growth forms include lianas, shrubs, vines, epiphytes, and herbs. The last of these, the herbs, form a varied group with a rich diversity of ferns, monocots and dicots all represented. Understory herbs are ecologically important and may, depending on forest structure and ecological conditions, make up 10–20% of the total tropical forest floras (Linares-Palomino et al., 2009; Cicuzza et al., 2013). Although there are published studies discussing the variation of species richness and community composition of tropical forest herbs at local to regional scales (e.g.,  Poulsen & Balslev, 1991; Annaselvan & Parthasarathy, 1999; Chittibabu & Parthasarathy, 2000; Costa, Magnusson & Luizao, 2005; Linares-Palomino et al., 2009; Cicuzza et al., 2013; Gómez-Díaz et al., 2017), these studies typically lump diverse functional groups of forest herbs differing in morphological, architectural, and ecophysiological traits without considering how these traits may relate to their co-occurrence.

Tropical forest herbs are themselves a varied group with considerable phylogenetic and ecological diversity. Among these, broad-leaved terrestrial monocot herbs are widespread and significant ecological components of the shaded understories of wet neotropical forests as well as in higher light environments of large forest gaps and forest edges. Seven families comprise the great majority of these herbs in the neotropics—the Araceae, Costaceae, Cyclanthaceae, Heliconiaceae, Marantaceae, and Zingiberaceae. With the exception of the Cyclanthaceae and addition of the Musaceae, these same families likewise form much of the understory community of broad-leaved monocot herbs in paleotropical forests. Despite their abundance and widespread coverage in the understory and forest edges of wet tropical forests, relatively limited attention has been given to the ecological evolutionary significance seen in patterns of life history traits of these species.

Two functional groups of broad-leaved terrestrial monocot herbs can be recognized based on their broad niche structure related to light availability. One group is formed by herbs restricted to the low light conditions in the shaded understory of primary forest habitats with only infrequent and short-term exposure to sunflecks (Björkman & Ludlow, 1972; Chazdon & Fetcher, 1984; Chazdon, 1986b; Chazdon, 1988; Le Gouallec, Cornic & Blanc, 1990). Ambient light levels are below 1% of full sun for the great majority of the day but irregularly impacted by brief sunflecks. The second group of species of broad-leaved herbs are found in higher light environments of forest edges along roads, road trails and developed areas, as well large gaps, where they receive sunflecks of high irradiance for limited but significant periods of the day (Galo, Rich & Ewel, 1992; Rundel et al., 1998).

In recent decades ecologists have suggested that functional traits of species could predict community response to environmental change and the effects of changes in community composition on ecosystem processes (Lavorel et al., 1997; Chapin et al., 2000; Díaz & Cabido, 2001). A conceptual framework, distinguishing traits that predict how species respond to environmental factors (response traits) from those traits that affect ecosystem processes (effect traits), was developed by Lavorel & Garnier (2002). These authors posited that the use of species traits, rather than individual species identity, to understand and predict community processes was a ‘Holy Grail’ in ecology (Funk et al., 2017). The developing concept of the leaf economic spectrum (LES) in recent years has spurred an increased attention to trait-based methodological approaches. The LES established relationships among several key traits across a broad range of species and different climate regimes (Reich, Walters & Ellsworth, 1997; Wright et al., 2005), and suggested that simple predictors such as specific leaf area and leaf nitrogen content may link to complex ecological processes such as growth rate and productivity. This idea was complicated by Osnas et al. (2013), who found that much of the correlation reported by Wright et al. (2005) was a result of normalizing trait data by leaf mass. After removing the effects of normalization, trait correlations were more complex and multi-dimensional (Osnas et al., 2013).

Ecologists are still working towards an understanding of how and why functional traits are correlated with each other (Díaz et al., 2016) and with community- and ecosystem-level processes (Griffin-Nolan et al., 2018; Yang, Cao & Swenson, 2018). A major unanswered question is to what extent patterns of functional trait variation are predicted by ecological variables versus by phylogenetic relatedness (Losos & Miles, 1994). The first hypothesis emphasizes the efficacy of natural selection in fitting traits to the local environment, while the second hypothesis raises the possibility that communities may be shaped largely by historical contingency and evolutionary constraint.

We investigate broad patterns of ecology, morphology, and functional traits in the flora of a lowland wet tropical forest at the La Selva Biological Station in Costa Rica. We focus on two ecological groups of broad-leaved terrestrial monocot herbs: understory (low-light) versus gap and forest-edge (high-light). We compare and contrast morphological and life history traits (plant architecture, leaf size and specific leaf area, leaf nitrogen content, leaf water use efficiency, and modes of vegetative reproduction) for 40 species from six different families.

## Material and Methods

**Figure 1:**
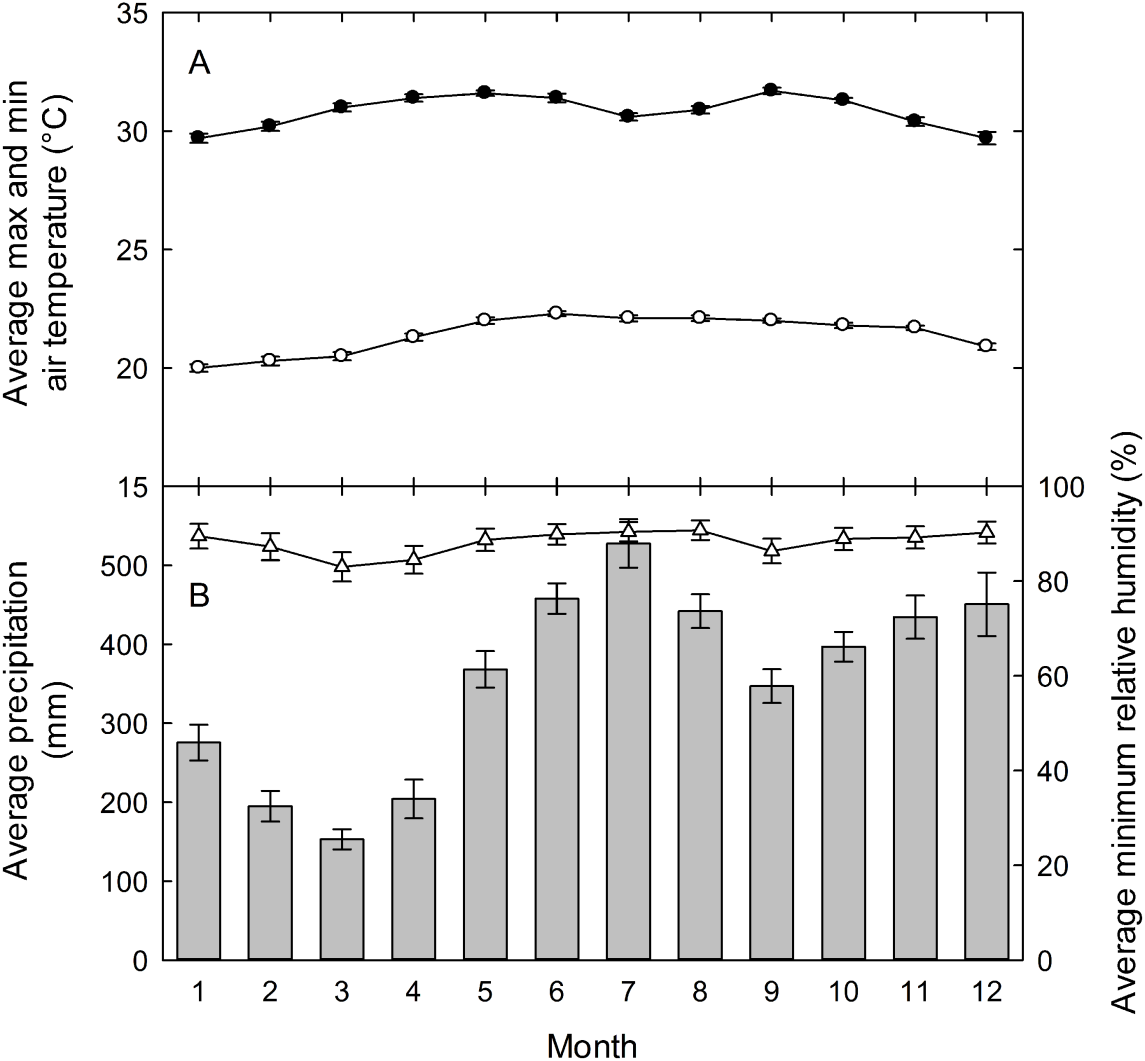
La Selva climate diagram. (A) Mean daily maximum (closed circles) and minimum (open circles) air temperatures, and (B) total monthly precipitation (vertical bars) and mean daily minimum relative humidity (open triangles) (B) for the La Selva Biological Station, Costa Rica. Values are means ± standard errors for each month for data collected hourly from 1957 to 2003 for precipitation, from 1982 to 2003 for temperature, and from 1992 to 2003 for relative humidity. Graph courtesy of Eric Graham.

Field studies on understory herbs were conducted at the La Selva Biological Station from 2000 to 2009. La Selva is a 1500-hectare reserve of premontane wet forest in the Atlantic lowlands of Costa Rica (10°28′N, 83°59′W). The research station has a mean annual rainfall of 4,244 mm (1958-2004), with mean monthly rainfall above 300 mm from May through December (Fig. 1). There are peaks of precipitation above 400 mm mo^−1^ in June-August and November-December, and a drier period from January through April (Cooley, Reich & Rundel, 2004). Even in the driest period of February and March, however, rainfall averages above 150 mm each month. Mean monthly maximum and minimum temperatures show little seasonal change, with mean highs of 30−31° C each month and mean monthly lows ranging only from 20−22° C.

**Figure 2:**
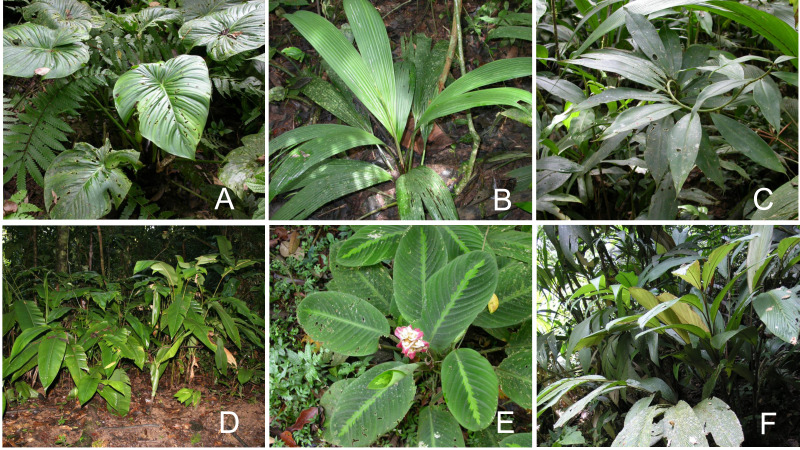
Understory herbs. Understory monocot herbs. (A) *Philodendron grandipes* (Araceae); (B) *Costus scaber* (Costaceae); (C) *Asplundia uncinata* (Cyclanthaceae); (D) *Heliconia irrasa* (Heliconiaceae); (E) *Goeppertia leucostachys* (Marantaceae); and (F) *Renealmia pluriplicata* (Zingiberaceae). Photos by Jennifer Sun.

**Figure 3:**
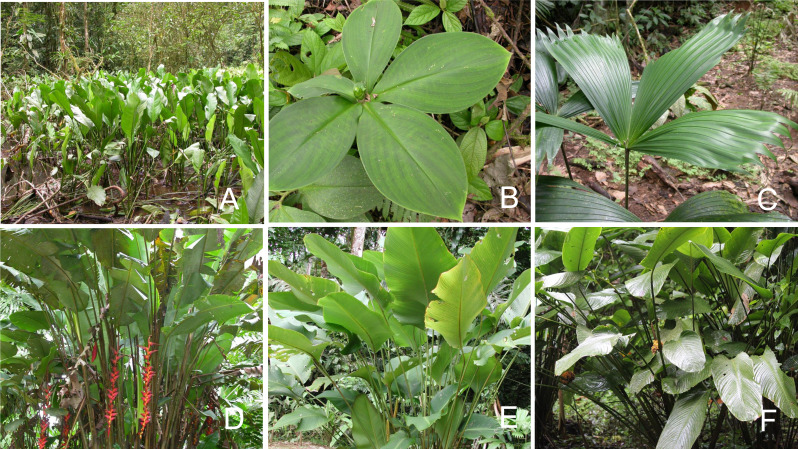
Forest edge herbs. Forest edge monocot herbs. (A) *Spathiphyllum friedrichsthallii* (Araceae); (B) *Costus malortieanus* (Costaceae); (C) *Carludovica sulcata* (Cyclanthaceae): (D) *Heliconia pogonantha* (Heliconiaceae); (E) *Calathea crotalifera* (Marantaceae); and (F) *Goeppertia inocephala* (Marantaceae). Photos by Jennifer Sun.

We restricted our study to six significant families—Araceae, Cyclanthaceae, Costaceae, Heliconiaceae, Marantaceae, and Zingiberaceae (Figs. 2 and 3). Field experience allowed us to familiarize ourselves with the natural history and ecological occurrence of 21 species of broad-leaved terrestrial understory monocots and 19 species characteristic of forest edges and/or moderate to large gaps which experience significant daily periods of high light irradiance. Our study species included all of the common understory and forest edge taxa as well as selected rarer taxa with interesting ecology (Table 1). These two groups collectively comprised more than half of the 78 species of terrestrial broad-leaved monocot herbs present in these families at La Selva (Wilbur & Collaborators, 2002).

**Table 1:** Morphological and canopy traits by species. Habitat, occurrence, and means of canopy and leaf morphological traits of understory and forest edge broad-leaved monocot herbs at the La Selva Biological Station, Costa Rica. Comparative data on understory palms are added at the end of the table.

Understory	Occurrence	Habitat specialization	Leaf arrangement	Height (m)	No. of leaves	Leaf area (cm2)	Canopy leaf area (m2)
**Araceae**							
*Anthurium ochranthum* C. Koch	**I**	Alluvial/wet	Basal	0.77	3.6	856	0.31
*Dieffenbachia longispatha* Engl. & K. Krause	A	Alluvial/wet	Basal	1.81	8.0	1968	1.57
*D. tonduzii* Croat & Grayum	LC	Alluvial/wet	Basal	0.77	7.8	492	0.38
*Philodendron grandipes* K. Krause	A	Alluvial/wet	Basal	0.64	8.0	675	0.54
*Spathiphyllum fulvovirens* Schott	A		Basal	0.76	9.3	436	0.41
*S. laeve* Engl.	I		Basal	0.74	11.8	233	0.27
*S. phryniifolium* Schott	I		Basal	1.64	13.8	1280	1.77
*S. wendlandii* Schott	LC	Alluvial/wet	Basal	0.79	16.5	785	1.30
**Costaceae**							
*Costus scaber* Ruiz & Pav.	A		Cauline	1.87	79.7	172	1.37
**Cyclanthaceae**							
*Asplundia sleeperae* Grayum & Hammel	I		Basal	1.87	14.2	3353	4.72
*A. uncinata* Harling	A		Basal	1.13	10.8	1346	1.45
**Heliconiaceae**							
*Heliconia irrasa* Lane ex R.R. Sm.	A		Basal	1.14	6.9	370	0.25
*H. umbrophila* G.S.Daniels & F.G. Stiles	I		Basal	1.98	12.7	1027	1.30
**Marantaceae**							
*Goeppertia cleistantha (* Standl.) Borchs. & S. Suárez	A	Alluvial/wet	Basal	0.48	6.1	344	0.21
*G. gymnocarpa* (H. Kenn.) Borchs. & S. Suárez	LC		Basal	1.43	31.4	1080	3.39
*G. leucostachys* (Hook. f.) Borchs. & S. Suárez	LC		Basal	0.54	12.1	434	0.52
*G. micans* (Mathieu) Borchs. & S. Suárez	A		Basal	0.40	11.6	11	0.01
*G. warscewiczii* (Mathieu) Borchs. & S. Suárez	I		Cauline	1.13	20.3	291	0.59
*Ischnosiphon inflatus* L. Andersson	I		Branched	1.84	38.9	638	2.48
**Zingiberaceae**							
*Renealmia cernua* (Sw.) J.F. Macbr.	A		Cauline	2.20	74.9	242	1.81
*R. pluriplicata* Maas	I		Cauline	1.18	25.6	484	1.24
**Mean**				**1.20**	**20.19**	**787**	**1.27**
**Standard deviation**				**0,56**	**20.9**	**753**	**1.12**
**FOREST EDGE AND LARGE GAP**							
**Araceae**							
*Spathiphyllum friedrichsthallii* Schott	LC	Open bog	Basal	1.55	7.2	937	0.67
**Costaceae**							
*Costus malortieanus* H. Wendl.	A		Cauline	0.76	5.4	264	0.14
*C. laevis* Ruiz & Pav.	LC	Alluvial soils	Cauline	4.58	148	610	9.02
**Cyclanthaceae**							
*Carludovica sulcata* Hammel	A		Basal	3.50	5.6	259	2.98
*Cyclanthus bipartitus* Poit.	A		Basal	2.12	8.5	2235	1.90
*Dicranopygium wedelii*	LC	Rheophyte	Basal	0.84	14.4	270	0.39
**Heliconiaceae**							
*Heliconia imbricata* (Kuntze) Baker	A		Basal (S)	5.25	11.1	8054	8.94
*H. latispatha* Benth.	A	Alluvial/wet	Basal (S)	3.97	9.5	3454	4.49
*H. mathiasiae* G.S. Daniels & F.G. Stiles	A		Cauline	3.65	34.0	781	2.66
*H. pogonantha* Cufod.	LC		Basal (S)	4.62	13.6	6957	9.46
*H. sarapiquensis* G.S. Daniels & F.G. Stiles	R		Basal (S)	2.20	21.0	1704	3.58
*H. wagneriana* Peterson	LC		Basal (S)	3.71	13.6	4354	5.92
**Marantaceae**							
*Calathea crotalifera* S. Watson	LC		Basal (S)	3.64	16.2	3862	6.26
*C. lasiostachya* Donn. Sm*.*	I		Basal (S)	1.03	14.0	580	0.81
*C. lutea* (Aubl.) Schult	LC		Basal (S)	3.72	26.6	4628	12.31
*C. similis* H. Kenn.	R	Alluvial soils	Basal (S)	2.85	14.5	3114	4.52
*Goeppertia inocephala* (Kuntze) Borchs. & S. Suárez	LC		Basal	2.88	23.9	2990	7.15
*G. marantifolia* (Standl.) Borchs. & S. Suárez	A		Cauline	1.52	11.6	440	0.51
*Pleiostachya leiostachya* (Donn. S.) Hammel	A	Alluvial soils	Basal(S)	3.20	9.00	2326	2.09
**Mean**				**2.93**	**21.5**	**2517**	4.41
**Standard deviation**				**1.34**	**31.5**	**2294**	**3.63**
**UNDERSTORY PALMS**							
*Asteogyne martiana* (H. Wendl.) H. Wendl. ex Hemsl.	A		Basal	1.50	21.3	1412	3
*Geonoma congesta* H. Wendl. ex Spruce	A		Basal	5.67	9.00	1917	1.72
*G. cuneata* H. Wendl. ex Spruce	A		Basal	1.17	11.1	1261	1.4

**Notes.**

A abundant LC locally common I infrequent r rare

Each species was characteristically found in only one of the two light environments. For five of the families, however, we were able to identify both understory and forest-edge species. This phylogenetic structure allows us to assess the degree to which functional traits covary with ecological or selective factors versus phylogeny.

All but one of the nine species of Araceae sampled were shade-tolerant understory species in the genera *Anthurium, Dieffenbachia, Philodendron,* and *Spathiphyllum*. The bog plant *S. friedrichsthallii* was the only open site member of the family. The Costaceae was represented by one forest understory species and two forest edge species of *Costus*. The Cyclanthaceae was represented by two terrestrial species of *Asplundia* in the forest understory and a single species each of *Carludovica, Cyclanthus,* and *Dicranopygium* from forest edges. The Heliconiaceae studied included *Heliconia irrasa* and *H. umbrophila i* n the understory and six species growing at forest edges or open sites. The largest number of species in the study came from the Marantaceae which is well represented in both understory and forest edge habitats. Included were 11 species traditionally placed in *Calathea* but now split between *Calathea* s.s. and *Goeppertia* (Borchsenius, Suárez & Prince, 2012) and a single species each of *Ischnosiphon* and *Pleurostachya*. Finally, the Zingiberaceae was represented by two species of *Renealmia* from the forest understory.

We did not attempt to include terrestrial ferns and herbaceous monocots and dicots from other families in our surveys, as our goal was to characterize the convergence in morphology and adaptive traits of the most characteristic broad-leaved monocot groups. These terrestrial ferns and other herbaceous monocots may be relatively abundant in the understory of early successional forests but are generally a relatively minor component of the understory of old growth tropical forests. Although a common understory component, we excluded understory palms from consideration as they differ in a number of ways from these broad-leaved herbs, as discussed below, and are thus better considered as shrubs in examining their functional group (Chazdon, 1986a; Cooley, Reich & Rundel, 2004). There are broad-leaved monocot herbs present in other families where selection has led to broad-leaved morphologies, often occurring along forest edges. This trait can be seen in several species of Poaceae, with *Streptochaeta sodiroana* Hack., *Pharus latifolius* L., *P. virescens* Döll*,* and *P. vittatus* Lem. all exhibiting unusually broad leaves for grasses, typically 4–7 cm in width. In open early successional forests and along forest edges one can commonly find *Zebrina zanonia* (L.) Sw. (Commelinaceae) and *Xiphidium coeruleum* Aubl. (Haemodoraceae), both of which develop relatively broad leaves.

The selected broad-leaved monocot herbs were divided into two groups as described above (Table 1). For each of the species we described and quantified a variety of ecological, morphological, and life history traits. The relative abundance of each species was categorized on a simple scale of abundant, locally common, infrequent, and rare based on our overall field experience. Abundant indicates typical presence in most moderate to large local stands of forest, while locally common indicates less widespread occurrence but local abundance in favorable habitats. Infrequent indicates only occasionally encountered. Those species with strong habitat preferences for alluvial soils or open bogs were defined.

Leaf and canopy morphological traits were measured for each of the 40 study species. Two examples of the youngest fully mature leaves were collected from each of three individuals of these species. Measurements were specific leaf area, leaf water content (fresh weight minus dry weight over dry weight), and specific leaf weight (cm^2^ g^−1^). Leaf samples from three individual plants of each species were analyzed for nitrogen content. Canopy traits of five individuals of each species were measured for leaf arrangement (cauline, basal, or subbasal), number of leaves, total plant leaf area, plant height, and presence or absence of vegetative propagation.

Stable carbon isotope ratios (*δ*^13^C) were determined to give an integrated measure of water use efficient over the time period in which carbon was fixed in leaf construction. Methodology and instrumentation followed procedures described by Rundel et al. (2019). Values are expressed as negative ratios with lower (more negative) values indicating low water use efficiency while less negative numbers indicate higher water use efficiency (Ehleringer & Rundel, 1989). Gas exchange measurements were carried out using both a LI-COR 6400 gas exchange instruments (Li-Cor, Lincoln, Neb., USA as described by Rundel et al., 2019).

**Table 2:** Ecophysiological traits by species. Mean values of ecophysiological and reproductive traits of understory and forest edge broad-leaved monocot herbs at the La Selva Biological Station, Costa Rica. Comparative data on understory palms are added at the end of the table.

**FOREST UNDERSTORY**	Leaf water content (g g-1)	Specific leaf area (cm2 g-1)	Nitrogen (%)	N specific weight (mg m-2)	d13C (o/oo)	Pmax (umol m-2 s-1)	Vegetative reproduction
**Araceae**							
*Anthurium ochranthum*	**5.39**	233	3.94	1.51	−34.4		
*Dieffenbachia longispatha*	4.96	157	3.65	2.32	−34.4	2.03	Clonal
*D. tonduzii*	8.25	205	3.88	1.89	−32.7		Clonal
*Philodendron grandipes*	6.54	273	3.79	1.39	−36.0	2.06	Clonal
*Spathiphyllum fulvovirens*	4.99	191	4.65	2.43	−37.3	2.80	
*S. laeve*	4.24	191	4.33	2.27	−37.1		
*S. phryniifolium*	5.68	266	4.14	1.56	−38.6	2.81	
*S. wendlandii*	6.73	196	3.79	1.93	−38.5	2.89	
**Costaceae**							
*Costus scaber*	5.84	185	2.45	1.32	−34.8	1.60	Clonal
**Cyclanthaceae**							
*Asplundia sleeperae*	4.32	169	3.08	1.82	−38.2		
*A. uncinata* Harling	4.71	166	2.31	1.39	−38.3	2.17	Clonal
**Heliconiaceae**							
*Heliconia irrasa* Lane	4.90	260	2.62	1.01	−34.7	3.18	Clonal
*H. umbrophila*	5.39	243	3.87	1.59	−34.1	2.93	
**Marantaceae**							
*Goeppertia cleistantha*	6.08	296	3.59	1.21	−35.9		
*G. gymnocarpa*	5.93	223	3.34	1.50	−34.2		
*G. leucostachys*	10.43	260	2.42	0.93	−33.9		
*G. micans*	5.47	413	3.08	0.75	−37.3		Clonal
*G. warscewiczii*	8.72	312	3.21	1.03	−37.0		Clonal, nodal
*Ischnosiphon inflatus*	3.73	241	2.57	1.07	−35.8		Nodal rooting
**Zingiberaceae**							
*Renealmia cernua*	4.04	165	2.88	1.75	−35.4		
*R. pluriplicata*	4.75	212	2.12	1.00	−36.6	1.40	
**Mean**	**5.39**	**231**	**3.34**	**1.44**	**−36.0**	**2.39**	
**Standard deviation**	**1.64**	**61**	**0.72**	**0.48**	**1.73**	**0.61**	
**FOREST EDGE AND LARGE GAPS**							
**Araceae**							
*Spathiphyllum friedrichsthallii*	5.78	200	4.44	2.22	−35.8	7.11	Clonal
**Costaceae**							
*Costus malortieanus*	12.83	240	2.39	1.00	−34.8		Clonal
*C. laevis*	5.16	93					Clonal
**Cyclanthaceae**							
*Carludovica sulcata*	3.97	186	3.40	1.83	−36.4		Clonal
*Cyclanthus bipartitus* .	4.80	189	3.12	1.65	−36.2		
*Dicranopygium wedelii*	4.97	186	3.04	1.63	−32.2		Clonal
**Heliconiaceae**							
*Heliconia imbricata*	4.07	180	3.00	1.67	−32.3		Clonal
*H. latispatha*	4.26	206	4.15	2.01	−31.3	14.06	Clonal
*H. mathiasiae*	4.21	189	3.36	1.78	−34.2	8.21	Clonal
*H. pogonantha*	3.34	143	3.22	2.25	−28.4		Clonal
*H. sarapiquensis*	4.37	255	3.37	1.32	−35.5		Semi-clonal
*H. wagneriana*	1.84	93	3.25	3.49	−28.5		Clonal
**Marantaceae**							
*Calathea crotalifera*	3,23	168	3.02	1.80	−30.7		
*C. lasiostachya*	3.67	241	3.33	1.38		4.35	
*C. lutea*	3.34	177	2.92	1.65	−29.5	8.10	Clonal
*C. similis*	4.32	285	2.24	0.79	−36.4	6.85	
*Goeppertia inocephala*	4.33	200	2.70	1.35	−34.2		
*G. marantifolia*	5.56	311	3.26	1.05	−33.1		Nodal rooting
*Pleiostachya leiostachya*	3.85	210	3.25	1.55	−32.8		Clonal
**Mean**	**4.70**	**197**	**3.19**	**1.62**	**−33.1**	**8.11**	
**Standard deviation**	**2.22**	**55**	**0.52**	**0.60**	**2.7**	**3.23**	
**UNDERSTORY PALMS**							
*Asteogyne martiana*			2.05		−36.0	3.09	
*Geonoma congesta*							
*G. cuneata*			2.02		−34.3		

Trait means between understory and forest edge species were tested for significance using paired t-tests. Standard deviations of trait mean values for each group are shown in Tables 1 and 2. Raw data showing field data with means and standard deviations of measurements are shown in Tables S1, S2, and S3. Differences with a *p*-value of <0.05 were considered to be statistically significant. Linear regressions were plotted to examine the relationship of plant height to number of mature canopy leaves and total canopy leaf area for mean maximum rate of assimilation to leaf nitrogen content and specific leaf area (SLA).

Repeated observations of growth patterns over many months allowed us to evaluate the degree to which clonality was present in our study species. While almost all species showed some limited ability to vegetatively reproduce, there was a clear dichotomy between species with characteristic clonality from those in which vegetative reproduction was rare or not observed. This dichotomy is evident at the generic level in some of the understory Araceae. Species of *Dieffenbachia* such as *D. longispatha* and *D. toduzii* possess thick rhizomes that trail at or just below the soil surface and produce large clonal patches. In contrast, none of the four understory species of *Spathiphyllum* studied showed any significant vegetative reproduction. Similarly, a strong separation could be easily seen in clonal and non-clonal species of *Heliconia*.

## Results

### Relative species abundance

The understory flora of broad-leaved monocot herbs includes a mix of abundant, infrequent, and relatively rare species. Our qualitative classification ranked nine of our 21 study species as abundant, including at least one species in each of the six focal families (Table 1). These were *Dieffenbachia longispatha, Philodendron grandipes,* and *Spathiphyllum fulvovirens* (Araceae), *Costus scaber* (Costaceae), *Asplundia uncinata* (Cyclanthaceae), *Heliconia irrasa* (Heliconiaceae), *Goeppertia cleistantha* and *G. micans* (Marantaceae), and *Renealmia cernua* (Zingiberaceae).

We ranked eight species of forest edge and large gap habitats as abundant in our qualitative classification. These were *Costus malortieanus, Carludovica sulcata, Cyclanthus bipartitus, Heliconia imbricata, H. latispatha, H. mathiasiae, Goeppertia maranthifolia,* and *Pleiostachya leucostachya* (Table 1). Terrestrial species of Araceae were absent from these habitats with the exception of *Spathiphyllum friedrichsthallii*, an open bog specialist.

### Morphological, architectural, and ecophysiological, life history traits

Leaf arrangement between cauline and basal leaves had a strong phylogenetic link to family in most cases. All of the Araceae, Cyclanthaceae included in our study had basal leaf arrangements, while all of the Costaceae and Zingiberaceae had cauline leaves. One exception to all basal leaves was present in *Heliconia* with *H. mathiasiae* having cauline leaves. Most of the Marantaceae had basal or subbasal leaves but exceptions were *Goeppertia marantifolia* and *G. warscewiczii* with cauline and *Ischnosiphon inflatus* with a branched growth form.

In stature understory herbs were relatively short with a mean height of 1.2 m, driven in part by phylogenetic relationships. Three species of *Goeppertia*, the smallest understory species, were at or below 0.5 m in mean height, while six of the eight species of Araceae were below 0.8 m in mean height (Table 1). Relatively robust and taller species were also present in the understory with *Dieffenbachia longispatha, Asplundia sleeperae, Heliconia umbrophila,* and *Ischnosiphon inflatus* all averaging at least 1.8 m in height. Forest edge and gap species were on average 2.9 m in height, more than twice as tall as understory species with a number of species of *Heliconia* reaching to or above 4 m in height. Only *Costus malorieanus* and the rheophyte specialist *Dicranopygium wedelii* averaged less than 1 m in height.

**Figure 4:**
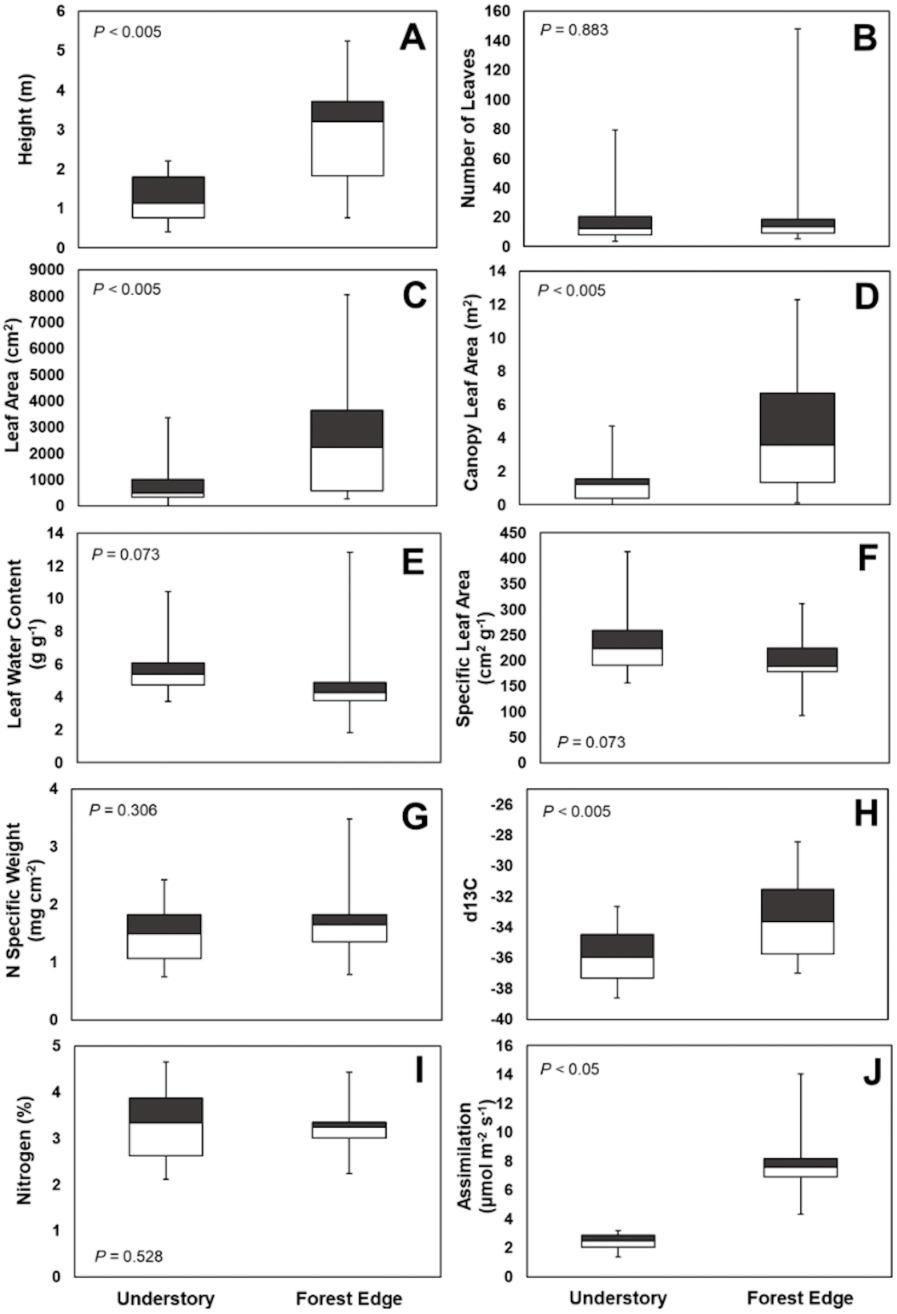
Trait comparisons. Habitat, abundance, canopy and leaf morphological traits (A, B, C, D) and ecophysiological traits (E, F, G, H, I and J) of understory and forest edge broad-leaved monocot herbs at the La Selva Biological Station, Costa Rica. Box graphs show the mean trait value, lowest and highest value, distribution of the minimum values (25th percentiles, lower boundary of box) and maximum value of the traits (75th percentiles upper boundary of box).

**Figure 5:**
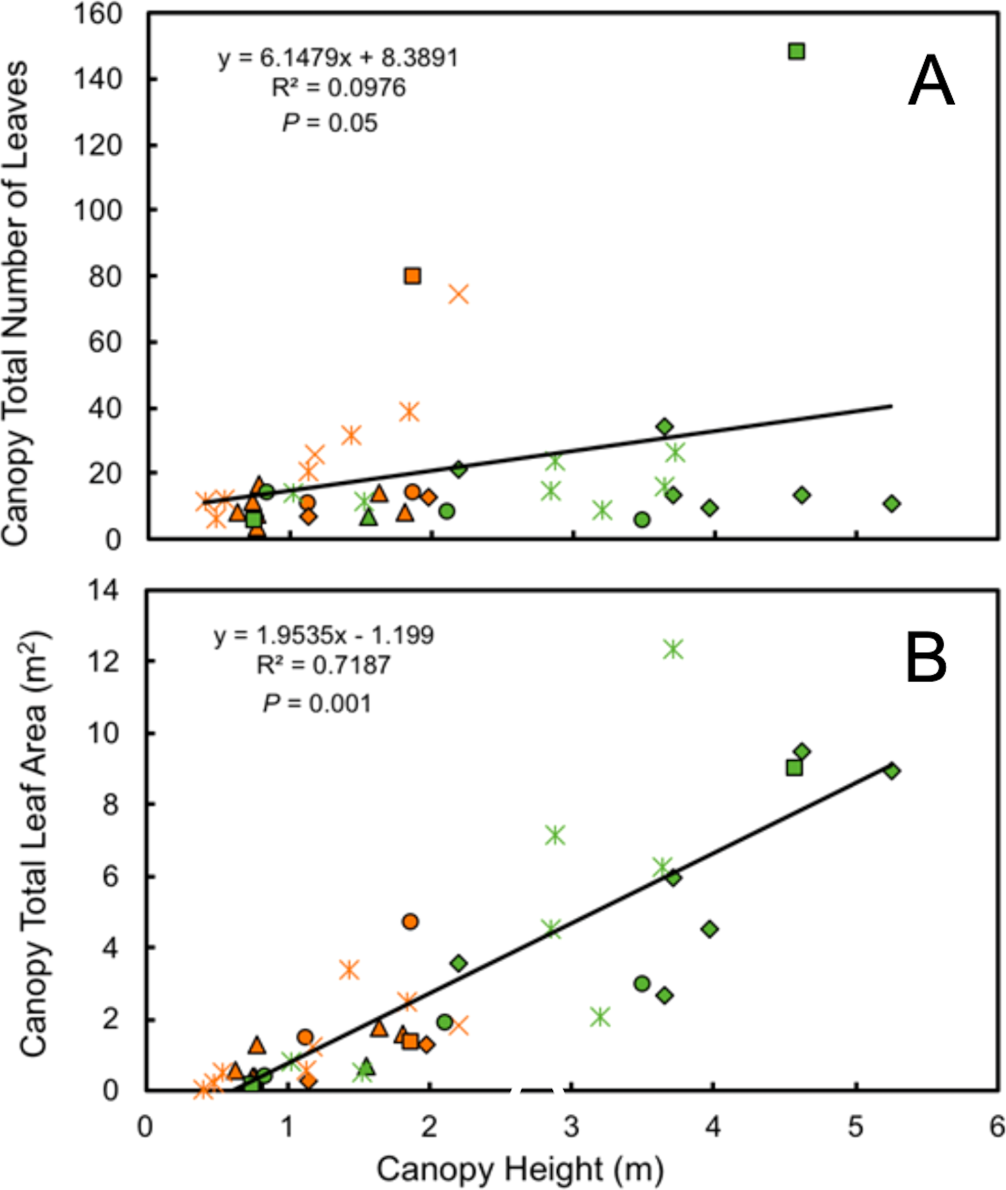
Morphological traits relatioships. Relationship of plant height to number of mature canopy leaves (A) and total canopy leaf area (B) for understory (orange symbols) and forest edge (green symbols) broad-leaved monocot herbs . Species families are identified by symbols as follows: triangle—Araceae, square—Costaceae, oval—Cyclanthaceae, diamond—Heliconiaceae, star—Marantaceae, x—Zingiberaceae.

Leaf number on mature plants was highly variable for both understory and forest edge species, due in part to canopy architecture. Despite the large difference in stature, the mean number of leaves per plant did not differ significantly between understory and forest edge and gap species, with 20.5 and 21.5 leaves, respectively (Fig. 4). Leaf number varied from a low of 3.6 in *Anthurium ochranthum* in the understory and 5.4 for *Costus malortieanus* at the forest edge to a high of 79.7 cauline leaves in the understory *Costus scaber* and 148 for the forest edge *Costus laevis.* Mean leaf number, although low in variability, was significantly related to plant height (Fig. 5).

Mean area of individual mature leaves was 787 cm^2^ for the understory species and three times greater at 2,517 cm^2^ for the forest edge species. The large leaf size of *Heliconia* and *Calathea* species heavily influenced to a high mean leaf area for this latter group. Again, however, the range of values for each group were highly variable. For forest understory species the sizes ranged from only 11 cm^2^ in *Goeppertia micans* to 1,968 cm^2^ in *Dieffenbachia longispatha*. The range for forest edge species was 264 cm^2^ for *Costus malortieanus* to 8,054 cm^2^ in *Heliconia latispatha.*

Total leaf area of individual plants paralleled the data for individual leaf size and was 3.5 times higher in the forest edge species but again with highly variable ranges of values. Mean total plant leaf area 1.23 m^2^ for understory herbs, ranging from only 0.01 m^2^ in the small *Goeppertia micans* to 4.72 m^2^ in *Asplundia sleeperae*. For forest edge species the mean plant leaf area was 4.25 m^2^, ranging from 0.14 m^2^ in *Costus malortieanus* to 12.31 m^2^ in *Calathea lutea*. Leaf canopy area was significantly related to plant height (Fig. 5).

Specific leaf area did not differ significantly between the two groups (Fig. 4). Understory species had a mean of 231 cm^2^g^−1^, ranging from 157 cm^2^g^−1^ in the thick leaves of *Dieffenbachia longispatha* to 413 cm^2^g^−1^ in *Goeppertia micans*. The mean SLA for forest edge taxa was 197 cm^2^g^−1^, with a low of 93 cm^2^g^−1^ in *Costus laevis* and *Heliconia wagneriana* and a high of 285 cm^2^g^−1^ in *Calathea similis*.

Despite the significant differences in leaf size (Fig. 4), leaf water content was not significantly different between the two groups, with individual species expressing a wide range of values. Understory species had a mean water content of 5.76 g g^−1^ with a range from 3.73 g g^−1^ in *Ischnosiphon inflatus* to 10.43 g g^−1^ in *Goeppertia leaucostachys*. Mean water content was 4.70 g g^−1^ for forest edge species with values from 1.84 g g^−1^ in *Heliconia wagneriana* to 12.83 g g^−1^ in *Costus malortieanus*.

Leaf nitrogen content also did not differ significantly between the two groups of species (Fig. 4) but showed some strong phylogenetic patterns. Understory species had a mean nitrogen content of 2.57%, with a low species mean of 2.31% in *Asplundia uncinata* and a high of 4.65% in *Spathiphyllum fulvovirens*. Mean values for forest edge species were similar in breadth from a low of 2,24% in *Calathea similis* to 4.44% in *Spathiphyllum friedrichsthallii*. Taken overall, Araceae tended to have higher than average nitrogen content while Marantaceae in both habitats tended to be lower than overall means.

Despite the relatively high leaf nitrogen contents of understory herb species, light-saturated rates of net photosynthetic assimilation were quite low. These ranged from 1.60 µmol m^−2^ s^−1^ in *Costus scaber* to 3.18 in *Heliconia irrasa*, with a mean of 2.39 µmol m^−2^ s^−1^ for the nine species measured. In contrast, rates for forest edge species ranged from maximum 4.35 µmol m^−2^ s^−1^ in *Calathea lasiostachya*, a gap species, to 14.09 µmol m^−2^ s^−1^ in *Heliconia latispatha* with a mean of 8.1 µmol m^−2^ s^−1^.

Mean values of stable carbon isotope ratio (*δ*^13^C) differed significantly between the understory and forest edge species reflecting their exposure to sun and relative humidity levels. The mean for understory species was -36.0 o/oo, with a range from -37.3 o/oo in *Spathiphyllum fulvovirens* and *Goeppertia micans* to -32.7 o/oo in *Dieffenbachia tanduzii*. The mean *δ*^13^C for forest edge species was -33.3 o/oo, ranging from -28.5 o/oo in *Heliconia wagneriana* to -36.4 o/oo in *Carludovica sulcata*.

## Discussion

Broad-leaved herbs in forest understories grow in an extremely low light environment where integrated light levels average only 1–2% of full sun. Even this low level exaggerates the typical conditions at the forest floor, where light levels are well below 1% of full sun much of the time and only occasional short-lived sunflecks bring more irradiance (Chazdon & Fetcher, 1984; Chazdon, 1988). Broad-leaved herbs growing in large gaps or forest edges, in contrast, experience a very different light environment, with direct solar radiation present for an extended period. Here we evaluate trait variation across two light environments and six families of broad-leaved monocot herbs.

### Ecology versus phylogeny

We find that the morphological and life history traits of the 40 species in this study reflect a combination of phylogenetic canalization and ecological selection for specific habitats. For example, most forest understory species are low-growing and basal in their arrangement of leaves, suggesting that this might be a habitat-specific trait. Exceptions to this pattern were present with phylogenetically clustered taxa, occurring with species of *Costus* and *Renealmia*. Both genera uniformly exhibited family traits of multiple smaller cauline leaves. These species also lie at the upper end of height for understory herbs (Table 1).

Under higher light conditions at forest edges and in large gaps, environmental selection is strong. Herb species characteristic of these habitats were tall and generally attained their heights by adopting a cauline or at least sub-basal form of canopy architecture. This is well illustrated in the cauline growth forms present in four out of seven species of forest edge and gap species of *Calathea* s.l., in contrast to the basal leaves of most understory species (Table 1). The mean height of the two understory species of *Heliconia* was 1.1 and 1.8 m, while all but one of the five high light species have mean heights above 3.3 m. The only relatively diminutive species in the latter group of species was *H. sarapiquensis* with a mean height of 2.2 m. This is a relatively rare species at La Selva and seems to grow in small gaps. Regardless of light environment, cauline leaves are generally much smaller than basal leaves in all of the broad-leaved monocot studied. These leaves had mean leaf areas of 80–537 cm^2^ in the nine species measured (Table 1).

**Figure 6:**
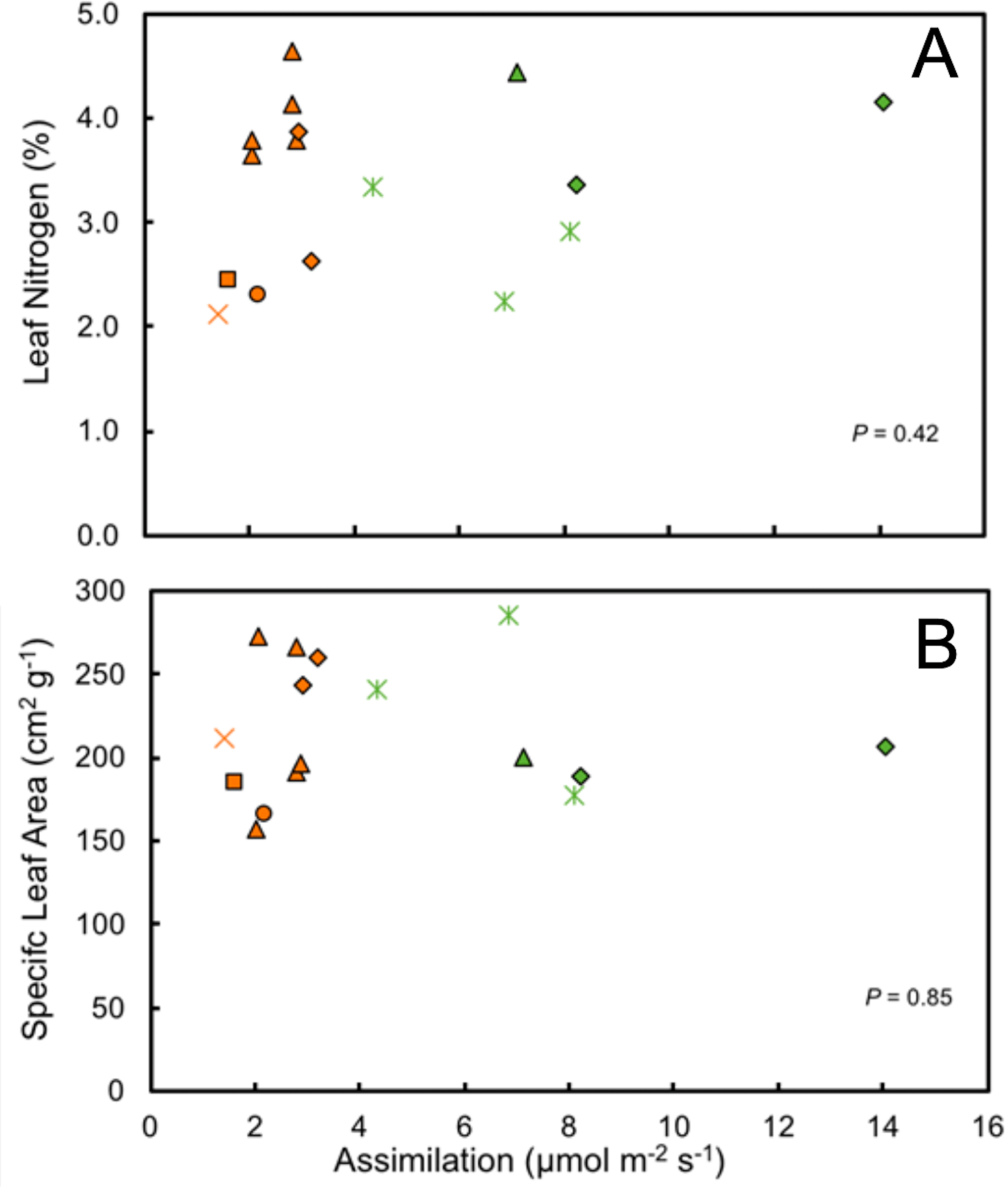
Ecophysiological trait comparisons. Relationship of mean maximum rate of assimilation to leaf nitrogen content (A) and specific leaf area (SLA) (B) for understory and forest edge broad-leaved monocot herbs. Species families are identified by symbols as follows:triangle—Araceae, square—Costaceae, oval—Cyclanthaceae, diamond—Heliconiaceae, star—Marantaceae, x—Zingiberaceae.

It is notable that leaf water content and specific leaf area were highly variable in the study species, exhibiting neither environmental nor phylogenetic patterns (Fig. 6). There were, however, elements of phylogenetic patterns in leaf nitrogen contents. All of the understory species of Araceae had leaf nitrogen contents above the average for all understory species (Table 2). There are a variety of ways in which leaf nitrogen concentration may indicate ecophysiological strategies related to relative nitrogen partitioning, the electron transport capacity per unit of chlorophyll, and/or the specific activity of RuBP carboxylase (Evans, 1989).

The photosynthetic capacity of understory herbs was low, despite high leaf nitrogen contents, with limited exposure to high levels of solar irradiance. Neither leaf nitrogen content nor specific leaf area showed any significant correlation with photosynthetic assimilation nor is a strong phylogenetic signal present (Fig. 6). Survival in shaded environments does not require maximizing photosynthetic capacity for a given leaf nitrogen content. Species that are well adapted to understory environments are known to partition relatively larger amounts of nitrogen into the thylakoids compared to sun species, a trait which produces lower photosynthetic capacity per unit of nitrogen (Evans, 1989). However, leaf nitrogen content and maximal rates of net photosynthetic assimilation among species do not scale well with photosynthetic capacity or leaf nitrogen content per unit leaf area. Photosynthetic rates, through their effects on growth, can strongly influence the population dynamics of plants in low and variable light environments, but the magnitude of this effect varies between species. In species in which fitness is independent of maximum rates of net photosynthesis there would be little opportunity for selection on photosynthetic rates (Westerband & Horvitz, 2017).

Although all of the species in this study exhibited at least some degree of vegetative reproduction, clonality is strongly developed in a number of species. This evolution of active vegetative reproduction does not show strong phylogenetic patterns, however, but rather a general but inconsistent relationship to habitat. For example, all of the forest edge species of *Heliconia* are strongly clonal, as is the understory species *H. irrasa*. However, the other understory *Heliconia*, *H. umbrophila*, is only weakly clonal. *Costus scaber* in the forest understory does not have active vegetative reproduction, while *C. malortieanus* and *C. laevis* growing along forest edges readily reproduce vegetatively from underground rhizomes.

### Adaptive tradeoffs

Why do understory herbs not grow taller, hypothetically exposing their leaves to a higher light environment? Clearly, there are “tradeoff” strategies to optimize fitness of these species. A competitive advantage accrues to herbs in open communities that grow taller and shade their close neighbors and capturing more light (Givnish, 1982). There is a selective tradeoff, however, as increased height requires reinforced structural tissues and thus a tradeoff in carbon allocation between height and support. In the low light environment of forest understories, there appears to be little selection to grow taller than heights of 0.5–2 m, perhaps because the added carbon gain associated with height beyond this level does not justify the added cost of structural tissues. Except in local areas where understory palms occur densely, understory herbs do not seem to strongly compete with light with their herb neighbors. In high light environments, however, there is a competitive advantage in greater height growth to overtop competitors (Valladares & Niinemets, 2008).

Another tradeoff exists in carbon allocation between structural tissues and photosynthetic tissues. This allocation is particularly critical in low-light understory environments where the benefit of a greater photosynthetic surface area requires carbon investment in structures to support that amount of biomass (Cooley, Reich & Rundel, 2004). For tropical understory palms at La Selva, support tissues have been shown to increase plant resilience to damage from falling debris, indicating tradeoffs between photosynthetic surface area and reinforcement of support tissues have been documented in Chazdon (1986a). Falling debris is a major cause of mortality in tropical understory plants including tree seedlings (Clark & Clark, 1989) and understory herbs (Gartner, 1989; Sharpe, 1993). Despite the ecological significance of falling debris, it has been shown that understory herbs do not exhibit the same degree of biomechanical strength in their petioles and stems as understory palms (Cooley, Reich & Rundel, 2004).

The biomechanical properties of *Asplundia uncinata* are intriguing in contrast to those of understory palms as this species is morphologically similar enough to the palms *Asterogyne martiana* and *Geonoma cuneata* to confuse a casual observer. Like these two understory palms, *Asplundia uncinata* occurs in large clonal clusters in deep shade, often excluding all other ground plants. Understory palm taxa form multi-ramet clones connected by thick aboveground rhizomes. Individual ramets exhibit slow and steady growth, attaining similar heights and displaying similar numbers of large bifid leaves. Despite obvious morphological similarities, *Asplundia uncinata* differs from the palms in the flexibility and strength of its petioles, with biomechanical traits that are like those of other understory herbs (Cooley, Reich & Rundel, 2004). These trait differences may reflect important growth strategies that enable the continued coexistence of morphologically similar species.

While the relative allocation of carbon to structural and photosynthetic tissues has obvious significance, there are clearly multiple successful strategies of arraying the architecture of photosynthetic surface area. Valladares, Skillman & Pearcy (2002) demonstrated this very clearly in a study of the efficiencies of light capture among 24 species of understory tree seedlings, low shrubs, and herbs representing a diversity of families, growth forms, and leaf attributes. Broad-leaved monocot herbs and other understory species showed convergence on a narrow range of light absorption efficiencies with ratios of absorbed light to available light of 0.60–0.75. Other studies have shown than that comparative growth by understory herbs and shrubs can be accomplished through alternative character syndromes, and leaf longevity may not be correlated with photosynthetic capacity in shade adapted plants related to leaf nitrogen content, SLA, and leaf longevity (Mulkey, Smith & Wright, 1991; Mulkey, Wright & Smith, 1992).

### Do functional traits predict species abundance?

Some species of broad-leaved terrestrial monocot herbs occur in great abundance while others are relatively rare. Can functional traits help explain their relative differences in abundance? Of the functional traits included in this study, none were predictive of species abundance. One possibility, of course, is that there are other critical functional attributes of ecophysiological limiting factors or reproductive traits that we were not measuring (Horwitz, 1993; Westerband & Horvitz, 2015; Tokarz et al., 2019). The suggestion has been made that there may exist a pool of functionally equivalent associated species present in a community confer resilience in ecosystem function (Walker, Kinzig & Langridge, 1999).

## Conclusions

The morphological and life history traits of broad-leaved monocot herbs demonstrate a mixture of phylogenetic canalization and ecological selection for specific habitats. Plant height, mean leaf area, canopy leaf area, *δ*^13^C, and net photosynthesis rates all differed significantly between these two habitats, consistent with the evolution of repeated adaptive shifts across multiple families. Clustering by phylogenetic relatedness, meanwhile, was observed for plant height, leaf arrangement, and leaf nitrogen content. The low stature of understory plants, compared to their close relatives from high-light environments, may reflect a lack of competition for light with their immediate neighbors, or may be driven by tradeoffs between height and structural reinforcement. Some traits, particularly leaf water content and specific leaf area, showed no obvious patterns by either evolutionary relatedness or light environment with the limited number of samples included. The evolution of these traits may be driven by environmental factors not considered here. Functional variation in measured traits was not correlated with species abundance in either habitat.

##  Supplemental Information

10.7717/peerj.9958/supp-1 Table S1 Raw data for leaf area, fresh weight, dry weight, specfic leaf area, and water content See Methods for sampling details and Table 1 for full species names.

10.7717/peerj.9958/supp-2 Table S2 Raw data Leaf N, d13C, d15N, and C to N ratio.

10.7717/peerj.9958/supp-3 Table S3 Raw data CO_2_ Assimilation (umol m-2 s-1) for understory and forest edge broad-leaved monocot herbs at the La Selva Biological Station, Costa Rica. Comparative data on understory palms are added. Replicated measurements of light response curves for photosynthetic assimilation were made for selected understory and forest edge species to determine maximum rates under light-saturating conditions. Gas exchange measurements were carried out using a LI-COR 6400 gas exchange instruments (Li-Cor, Lincoln, Neb., USA). Photosynthetic assimilation and stomatal conductance were measured in mid-morning and early afternoon over several days.

## Additional Information and Declarations

### Field Study Permissions

The following information was supplied relating to field study approvals (i.e., approving body and any reference numbers): Field work was carried out at the La Selva Biological Station, Costa Rica, with all research permits approved by MINAE in the Costa Rican government.

### Data Availability

The following information was supplied regarding data availability: Raw data are available as Supplemental Files.
